# Successful Management of Cervical Kyphosis With Myelopathy Across Multiple Vertebral Levels Using Microendoscopic Laminectomy: A Case Report

**DOI:** 10.7759/cureus.78302

**Published:** 2025-01-31

**Authors:** Yoshinori Maki, Keita Kuraishi, Yoshihiko Ioroi, Tamaki Kobayashi, Toshinari Kawasaki

**Affiliations:** 1 Neurosurgery, Hikone Chuo Hospital, Hikone, JPN; 2 Spinal Surgery, Ohmi Sebone Clinic, Ohmihachiman, JPN; 3 Neurosurgery, Kyoto Katsura Hospital, Kyoto, JPN; 4 Spinal Surgery, Kyoto Katsura Hospital, Kyoto, JPN

**Keywords:** cervical laminoplasty, cervical myelopathy, cervical spondylosis, less invasive surgery, microendoscopic surgery

## Abstract

Cervical spondylosis can result in myelopathy, manifesting as sensory and/or motor disturbances in the neck, upper extremities, and lower extremities. Conservative treatment, including medication and rehabilitation, can successfully manage the symptoms. However, surgical treatment is necessary when conservative treatment is not effective. Cervical laminectomy, laminoplasty, and fixation surgery, if required, are typical management for patients with cervical myelopathy. In daily practice, we sometimes encounter patients who do not wish for open surgery because of invasiveness. Here, we report a case of myelopathy with kyphosis related to cervical spondylosis in multiple vertebral levels successfully treated with microendoscopic laminectomy. A 68-year-old woman was bothered by myelopathy related to cervical kyphotic spondylosis. Medication did not resolve the myelopathic pain, which had lasted for four years. Radiological examination revealed cervical kyphosis and spinal cord compression from C2 to C4, and decompression and fixation surgery seemed warranted. However, the patient did not wish for open surgery. Thus, we performed microendoscopic laminectomy from C2 to C4. The patient returned home the day after surgery, and the pain resolved postoperatively. No complications occurred. Preoperative kyphosis and cervical alignment improved nine months after surgery. Microendoscopic laminectomy can successfully resolve myelopathy related to cervical spondylosis. This surgical approach appears to be a good option for patients who prefer not to undergo typical open surgery (e.g., conventional laminectomy or laminoplasty). The muscle structure can be preserved due to the minimally invasive nature of the procedure, potentially preventing postoperative kyphosis. This topic warrants further research.

## Introduction

Cervical spondylotic myelopathy is a common age-related disease that affects patients aged more than 55 years old [1]. This entity can result from intervertebral disk degeneration, osteophytes of facet joints, and ossification and hypertrophy of the ligamentum flavum and posterior longitudinal ligament [1]. While conservative management, including medications, neck immobilization, traction, and physical therapy, can resolve manifestations related to cervical spondylotic myelopathy, surgical treatment can be required for patients with severe neurological symptoms [1].

Open cervical laminoplasty (CLP) is a standard treatment for cervical spondylotic myelopathy [2-4]. Although this posterior approach can effectively resolve cervical spondylotic myelopathy, the nuchal muscle can be damaged and become atrophic [5]. CLP with a small skin incision is proposed to reduce the method's invasiveness to the posterior anatomical structures [5]. However, persistent neck pain and/or stiffness remain major postoperative complications [5].

Cervical microendoscopic laminoplasty (CMEL), initially proposed by Minamide et al., is an even less invasive surgical approach for cervical spondylotic myelopathy [6]. Postoperative satisfactory preservation of the cervical alignment in patients undergoing CMEL compared to those undergoing CLP was reported [6-9]. This endoscopic operation can be performed for single- or multi-level cervical spondylotic myelopathy. However, CMEL is not generally applied to cases of cervical spondylotic myelopathy accompanied by kyphosis, for which fixation surgery may be required.

In this paper, we describe a case of multi-level cervical myelopathy and concurrent kyphosis successfully treated with CMEL, suggesting that this minimally invasive procedure can also be an option for cervical myelopathy accompanied by kyphosis.

## Case presentation

**Figure 1:**
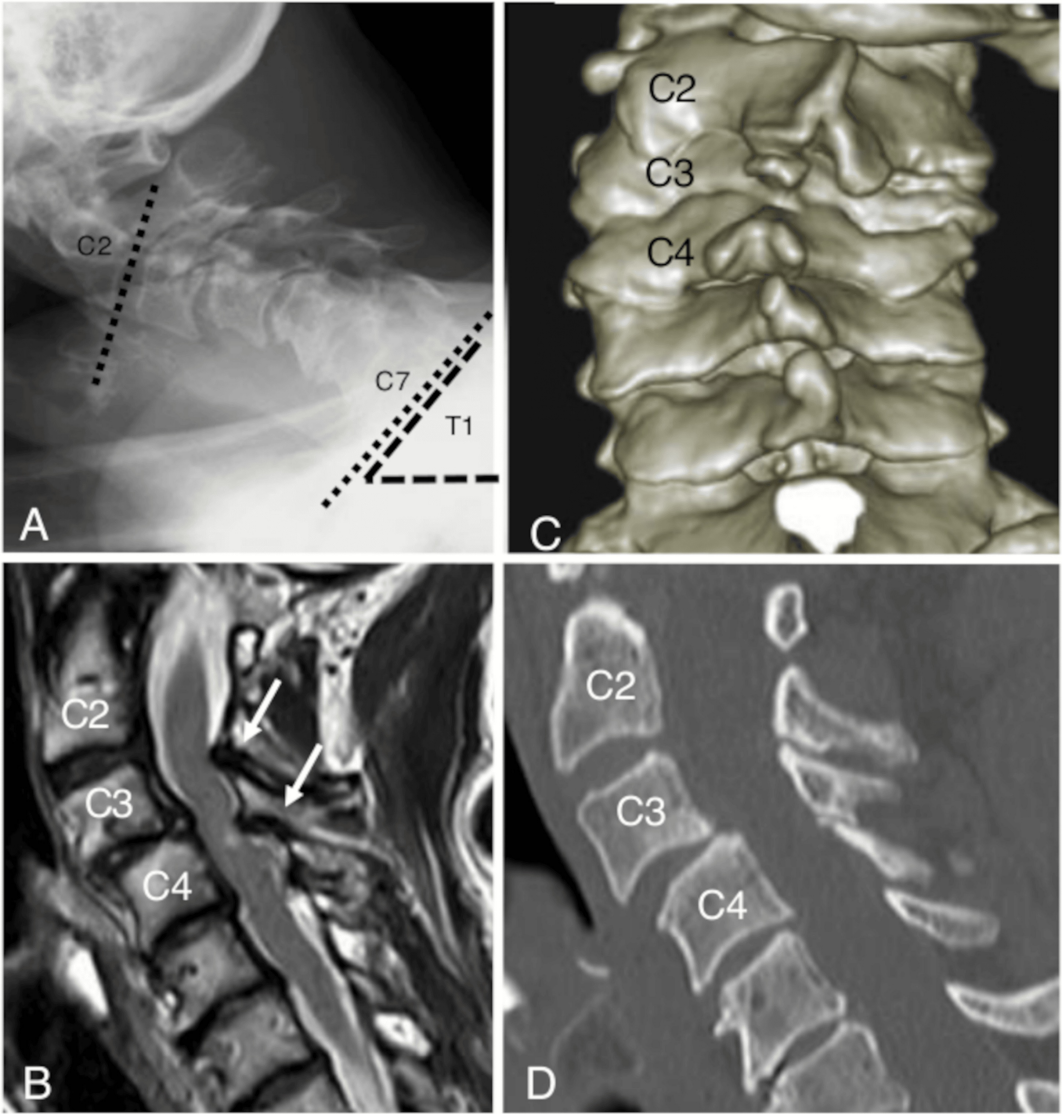
Preoperative images (A) On X-ray, the C2-C7 Cobb angle (dotted lines) and T1 slope (dashed lines) were -13° and 55°, respectively. (B) A sagittal magnetic resonance image revealed severe cervical canal stenosis from C2 to C4 (white arrows). The canal stenosis resulted in myelopathy at the same levels. (C, D) Anterior spondylolisthesis at C3/C4 and scoliotic change were concurrently diagnosed on plain and 3D reconstructed computed tomography images.

A 68-year-old woman visited our clinic, complaining of pain in the neck, back, and right upper and lower extremities, lasting for over four years. These symptoms were not controlled with medications. The pain was aggravated by extension, and the patient spontaneously fixed her neck in flexion. The neck was not completely fixed, and the patient was able to lift her neck against gravity. Suspecting cervical myelopathy, we performed radiological examinations. X-ray showed a C2-C7 Cobb angle of -13° and a T1 slope of 55°. Anterior spondylolisthesis at C3/C4 and scoliotic changes were concurrently diagnosed on 3D reconstructed computed tomography images. Magnetic resonance imaging revealed cervical canal stenosis from C2 to C4 (Figure 1). The preoperative scores on the Japan Orthopaedic Association cervical myelopathy evaluation questionnaire and the visual analog scale were 10.5 and 6, respectively.

We believed that the patient's neurological symptoms might have resulted from these lesions. Initially, we proposed cervical posterior decompression and fixation surgery to the patient. However, the patient declined the proposal due to concerns about the invasiveness of the surgery and the limited neck movement that could follow fixation. After discussing the potential risks and the need for additional fixation surgery, and obtaining informed consent from the patient and her family, we planned CMEL for C2 to C4.

Operation

**Figure 2:**
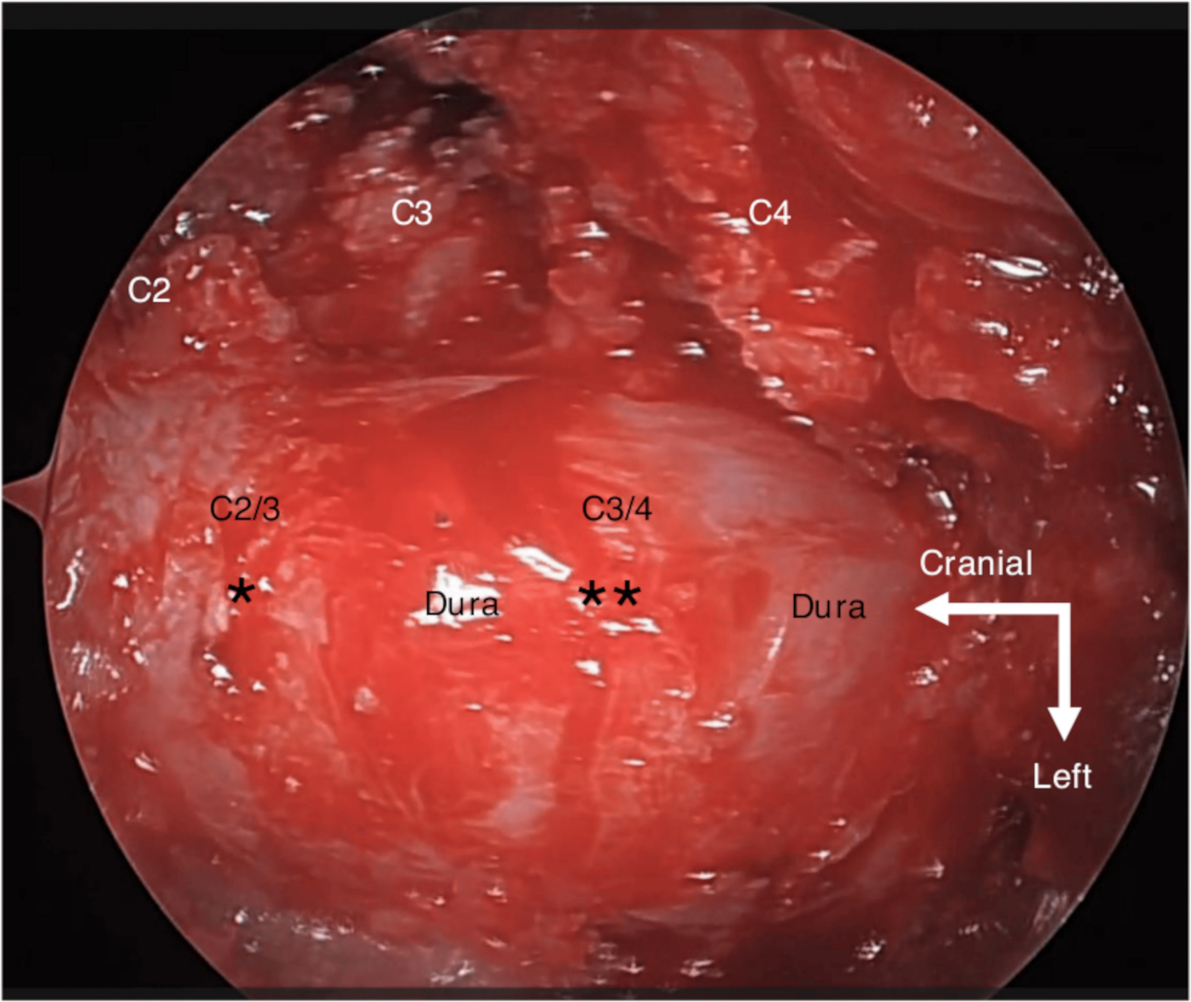
Intraoperative image of cervical endoscopic laminoplasty After decompression at C2-C3 and C3-C4 levels, the dura was exposed. The residual yellow ligaments indented the dura at C2/C3 (*) and C3/C4 (**) levels.

Under general anesthesia, the patient was positioned prone with the neck in flexion. C2-C4 vertebrae were confirmed under fluoroscopy. A midline skin incision of approximately 18 mm was made. The left paravertebral muscle at C2-C4 was detached using an electric knife, and the medial side of the facets from C2/C3 to C3/C4 was exposed. The nuchal ligament was preserved. A 16-mm short tubular retractor (METRx, Medtronic, Minneapolis, MN) was inserted to obtain the surgical field. Hemilaminectomy from C2 to C3 was followed by left cranial partial laminectomy of C4 and right medial partial laminectomy of C3 and C4. The ligamentum flavum and epidural fat from C2 to C4 were also removed (Figure 2).

After we decompressed the dura and achieved hemostasis using a low-power bipolar and microfibrillar collagen hemostat (Avitene, Becton, Dickinson and Company: BD), a UK drain catheter of 10Fr (NIPRO) was placed in the extradural space. The wound was closed.

Postoperative course

**Figure 3:**
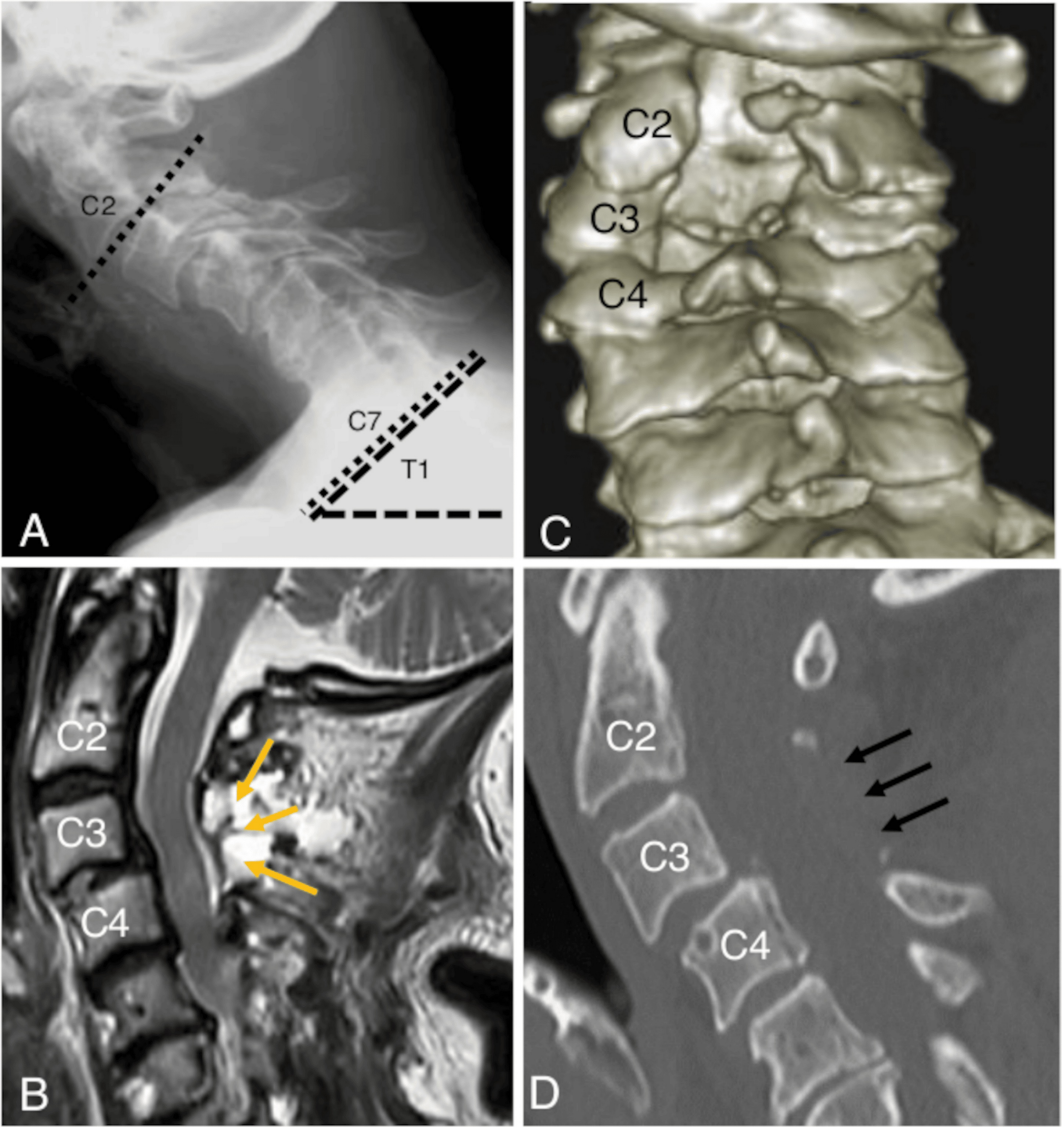
Postoperative images (A) Nine months after surgery, an X-ray showed the C2-C7 Cobb angle (dotted lines) and T1 slope (dashed lines) as -5° and 42°, respectively. (B) A follow-up magnetic resonance image revealed decompression of the spinal cord from C2 to C4 (yellow arrows). (C, D) Postoperative computed tomography images showed hemilaminectomy from C2 to C3 and partial laminectomy of C4 (black arrows).

The patient returned home 24 hours after surgery, the following day. She was advised not to get the insertion site of the drainage tube wet. A neck collar was prescribed to maintain the neck position. The drainage tube was removed two days later after confirming that the daily drained serum had decreased to below 30 mL. No complications occurred. The patient is now being followed in our clinic, and the preoperative pain has resolved. Cervical kyphosis gradually improved. The C2-C7 Cobb angle and T1 slope were -5° and 42°, respectively, on X-ray nine months after surgery (Figure 3). The postoperative scores on the Japan Orthopaedic Association cervical myelopathy evaluation questionnaire and the visual analog scale improved to 12.5 and 1, respectively. Even nine months after surgery, the patient still used the collar when necessary, as the cervical deformity had not completely resolved.

## Discussion

We presented a case of cervical myelopathy related to kyphosis and anterior spondylolisthesis managed by CMEL. The patient did not wish for open surgery initially but agreed with minimum invasive surgery. CMEL from C2 to C4 successfully resolved myelopathy. Consequently, kyphosis was not progressive. The postoperative course was satisfactory.

As for surgical management of cervical spondylotic myelopathy, CLP is an established and standard treatment [2-5]. However, compared to CMEL performed in our case, CLP has disadvantages such as a larger wound, postoperative axial pain, decreased cervical range of motion, and progressive kyphosis [4]. As a result, postoperatively, patients may need longer resting periods and neck collars. In addition, CLP provides a large posterior shift of the spinal cord, which can cause C5 paralysis [4]. Since CMEL is less invasive than CLP, especially in terms of postoperative pain and wound size, it is an acceptable surgical method even for patients who have psychological stress against CLP, as seen in our case.

The postoperative outcomes of CMEL are considered equivalent to those of CLP. In previous reports, CMEL has the following advantages: wound size, postoperative pain, postoperative cervical range of motion, C2-C7 angle, and less frequency of C5 paralysis occurrence [6-9]. Meanwhile, CLP seems suitable for those patients with a continuous type of ossification of the posterior longitudinal ligament (OPLL). Posterior decompression for multiple levels by CMEL in patients with a continuous type of OPLL requires experience, technique, and more operation time. In this regard, CMEL seems unfavorable, compared to CLP.

Generally, cervical kyphosis is considered contraindicated for posterior decompression surgery. Additionally, in our case, due to cervical kyphosis, posterior decompression surgery may not have been ideal. However, we proceeded with CMEL, as the patient did not wish to undergo CLP or fixation surgery. Before performing CMEL, we explained the potential need for additional fixation surgery if postoperative kyphosis were to progress. Postoperatively, cervical alignment improved, possibly because the spinal cord was adequately decompressed from the thickened yellow ligament. Minamide et al. mentioned that cervical lordosis could improve after CMEL [8]. Suda reported the impact of cervical malalignment on neurologic recovery after CLP for cervical spondylotic myelopathy; when patients have local kyphosis exceeding 13°, posterior decompression without correction of the kyphosis is not recommended [10]. The kyphosis in this case appeared more pronounced than those typically indicated for CLP or CMEL. However, postoperative alignment still improved, despite the detachment of the left paravertebral muscle at C2 from the vertebral body. This improvement could also be attributed to the well-preserved nuchal ligament using CMEL. Thus, CMEL may also be indicated for cervical myelopathy with concurrent kyphosis.

Limitations

This is a single case followed for a limited period after surgery. Therefore, long-term postoperative outcomes need to be further evaluated. In our case, CMEL was performed for two cervical levels. It remains unclear whether this procedure can be safely and effectively applied to three or more cervical levels or in cases of more severe kyphosis. With proper patient selection, the indications for CMEL could be expanded. Finally, it should also be evaluated whether this procedure can resolve cervical radiculopathy when combined with posterior nerve root decompression surgery (i.e., foraminotomy).

## Conclusions

We reported a case of cervical myelopathy resulting from kyphosis and anterior spondylolisthesis. CMEL from C2 to C4 effectively resolved the patient’s myelopathy. Because CMEL is less invasive, it can serve as an alternative management option for cervical myelopathy with concurrent kyphosis and spondylolisthesis. The utility of this method should be evaluated in more cases. If this surgical approach proves effective and safe enough to avoid fixation surgery, it could be applied to patients with high perioperative risks, such as those with advanced age or cardiopulmonary diseases. Further research is needed to establish evidence for the efficacy of CMEL in similar cases.
